# Influenza-Associated Hospitalizations in Unvaccinated Children Across Six Consecutive Seasons: Clinical Burden and Regional Vaccination Coverage Trends in Galați County, Romania

**DOI:** 10.3390/vaccines14060478

**Published:** 2026-05-28

**Authors:** Irina Profir, Cristina-Mihaela Popescu, Alexandru Nechifor, Mădălin Guliciuc, Ada Stefanescu

**Affiliations:** 1 Clinical Medical Department, Faculty of Medicine and Pharmacy, “Dunărea de Jos” University of Galați, 800216 Galați, Romania; irina.profir@ugal.ro; 2 “Sf. Ioan” Clinical Emergency Pediatric Hospital in Galați, 800487 Galați, Romania; 3 Medical and Pharmaceutical Research Center, Faculty of Medicine and Pharmacy, “Dunărea de Jos” University of Galați, 800008 Galați, Romania; alexandru.nechifor@ugal.ro (A.N.); madalin.guliciuc@ugal.ro (M.G.); ada.stefanescu@ugal.ro (A.S.); 4 Dental-Medicine Department, Faculty of Medicine and Pharmacy, “Dunărea de Jos” University of Galați, 800201 Galați, Romania; 5 Department of Individual Sports and Physiotherapy, Faculty of Physical Education and Sport, “Dunărea de Jos” University of Galați, 800008 Galați, Romania; 6 Department of Surgery, Faculty of Medicine and Pharmacy, “Dunărea de Jos” University of Galați, 800008 Galați, Romania; 7 Department of Urology, St. Apostol Andrei Emergency Clinical Hospital, 800578 Galați, Romania

**Keywords:** child, hospitalization, human influenza, infant, influenza vaccines, Romania, vaccination coverage

## Abstract

**Background:** Influenza vaccination coverage among children remains critically low in Romania, yet regional data contextualizing the clinical burden relative to local vaccine uptake are scarce. **Methods:** A retrospective descriptive study was conducted at “Sf. Ioan” Clinical Emergency Pediatric Hospital in Galați, Romania, including all patients aged 0–14 years with Reverse Transcriptase Polymerase Chain Reaction (RT-PCR)-confirmed influenza over six consecutive seasons (October 2019–April 2025). Regional influenza vaccination coverage data for children aged 0–14 years and pregnant women were obtained from county public health authority records and contextualized against Romanian National Institute of Statistics population data. **Results:** A total of 525 unvaccinated children were included. The median age was 2 years (IQR 1–5); 50.5% were aged 2 years or younger. Multisystem clinical involvement was documented in 97.52% of patients, with respiratory involvement predominating (86.66%). The median length of hospital stay was 5 days (IQR 4–7). Regional vaccination coverage never exceeded 2.0% of the eligible pediatric population in any study year, falling to 0.3% in 2023 and 0.1% in 2025. Infants aged 0–2 years were consistently the least vaccinated group (0–5 months not eligible for vaccination). Maternal vaccination was effectively absent in most seasons. **Conclusions:** These findings document a critical and persistent influenza vaccination gap among children and pregnant women in Galați County. Targeted public health interventions to improve vaccine uptake—particularly for infants, young children, and pregnant women—are urgently needed in this and similar low-coverage Eastern European settings.

## 1. Introduction

Influenza is an acute respiratory infection caused by influenza A and B viruses, transmitted primarily through respiratory droplets, aerosols, and contact with contaminated surfaces [1,2,3]. In children, illness typically presents with sudden-onset fever, cough, pharyngitis, nasal congestion, myalgia, and fatigue, although symptoms may overlap considerably with those of other respiratory viral infections [4]. Beyond uncomplicated respiratory illness, influenza can produce a broad spectrum of systemic manifestations—including lower respiratory tract involvement, gastrointestinal symptoms, hematological abnormalities, febrile seizures, and neurological complications such as encephalopathy—and even previously healthy children may develop severe disease necessitating hospitalization [5].

Globally, seasonal influenza is estimated to cause approximately one million hospitalizations annually in children under 5 years of age [6,7]. Young children, particularly infants under 2 years, are disproportionately affected due to immunological immaturity and limited prior antigen exposure. Annual vaccination remains the most effective preventive intervention. Both the American Academy of Pediatrics and the World Health Organization recommend universal influenza vaccination for all children aged 6 months and older, with additional emphasis on maternal vaccination during pregnancy as a strategy for indirect infant protection [8,9]. Despite these recommendations, influenza vaccine uptake among children remains suboptimal in countries with established vaccination programs. In the United States, coverage among children declined from approximately 57% in the 2019–2020 season to 47.8% by January 2024, continuing a post-pandemic downward trend [10,11,12].

In Europe, pediatric influenza vaccination coverage has remained persistently low and heterogeneous across multiple consecutive seasons. According to ECDC survey data covering the 2021–22 through 2023–24 influenza seasons, pediatric vaccination recommendations have progressively expanded—from 14 EU/EEA countries reporting influenza recommendations for children in the 2020–2021 season to all 30 EU/EEA countries recommending vaccination for children or adolescents in the 2024–2025 season—yet actual coverage rates among children have remained markedly below targets, ranging from 0.9% to 38.9% during the 2023–2024 season, with Eastern European countries including Romania consistently reporting some of the lowest rates on the continent [13].

In Romania, seasonal influenza vaccination is recommended for children aged 6 months and older under the National Vaccination Program, with administration in community pharmacies additionally authorized since 2022 to improve access [14]. National surveillance data document persistently low uptake across the full study period. In the 2019–2020 season—the final pre-pandemic season—only 0.3% of influenza vaccine doses were administered to children aged 6–59 months nationally, confirming that suboptimal pediatric coverage predated pandemic-era disruption [15]. Coverage remained equivalently low in 2020–2021 (0.3%), a period during which pandemic-related non-pharmaceutical interventions substantially altered vaccination program delivery across Romania and the broader European region [16]. In subsequent seasons, overall national influenza vaccination coverage across all recommended groups remained consistently below 10%—8.01% in 2021–2022, approximately 8% in 2022–2023, and 5.7% in 2023–2024—with pediatric uptake comprising only a small fraction of these totals [17,18,19]. Recent analyses confirm that influenza vaccines remain underutilized, particularly among children and pregnant women in Romania, despite official recommendations [20]. The COVID-19 pandemic additionally disrupted influenza circulation patterns, with near-complete suppression of influenza activity during 2020–2021 followed by marked post-pandemic rebounds in incidence, particularly among young children with limited prior antigen exposure [21,22].

Regional-level vaccination data from within Romania remain sparse in the published literature. National averages may mask substantial geographic heterogeneity in vaccine uptake, and pairing regional coverage data with hospitalization data from the same catchment area allows concurrent descriptive characterization of hospitalization patterns and regional vaccination coverage within the same geographic context, without implying causal or inferential relationships between the two data streams. This study addresses that gap by combining a six-season retrospective cohort of children hospitalized with RT-PCR-confirmed influenza at “Sf. Ioan” Clinical Emergency Pediatric Hospital, Galați, with county-level vaccination coverage data from the same catchment area. The study aimed to characterize the clinical and epidemiological profile of children hospitalized with RT-PCR-confirmed influenza and to contextualize the observed hospitalization burden against regional vaccination coverage trends across six consecutive influenza seasons (2019/20–2024/25), with the dual objective of providing actionable regional data to inform pediatric influenza vaccination policy.

## 2. Materials and Methods

### 2.1. Study Design and Setting

The retrospective descriptive study was conducted at “Sf. Ioan” Clinical Emergency Pediatric Hospital in Galați, the sole pediatric emergency hospital serving Galați County and the designated referral center for all pediatric inpatient care in the region. Children presenting with acute illness to smaller community health units within the county are systematically referred to this institution, ensuring that the hospital captures the totality of pediatric influenza hospitalizations requiring inpatient management within the catchment area. The institution operates at a secondary care level serving south-eastern Romania. The clinical component included children hospitalized with RT-PCR-confirmed influenza across six consecutive influenza seasons (October 2019–April 2025); the vaccination coverage component drew on county-level public health surveillance data for the same period. The study was performed and reported in accordance with the STROBE recommendations for observational studies.

Galați County, located in southeastern Romania, had a total resident population of 496,892 according to the 2021 national census. The pediatric population aged 0–14 years, the primary study population, ranged from 79,487 in 2019 to 73,858 in 2025, reflecting a progressive demographic decline consistent with broader regional depopulation trends documented across the study period (Romanian National Institute of Statistics—Institutul Național de Statistică—TEMPO online database). The single-center design, therefore, captures regional-level pediatric influenza hospitalization data with a high degree of completeness for this catchment area.

Access to identifiable electronic medical records was restricted to the primary researcher (I.P.) to ensure data consistency and maintain patient confidentiality. Following initial data extraction, a fully anonymized dataset was created by removing all direct personal identifiers. This anonymized dataset was subsequently made available to the complete author team for analysis and manuscript preparation.

In Romania, the influenza season typically spans weeks 40 through 20 of the calendar year (approximately October through May). According to ECDC and national surveillance reports, influenza activity generally rises in December, peaks between January and March, and declines by April–May [14,23]. These seasonal patterns were observed in the eastern region of the country, including Galați County, during the study period.

### 2.2. Clinical Cohort—Participants

All children aged 0–14 years hospitalized during the study period with RT-PCR-confirmed influenza were eligible for inclusion. Rapid antigen tests were used for initial clinical triage, with RT-PCR testing performed for confirmation in all included patients. Children hospitalized with influenza-like illness who lacked RT-PCR confirmation were excluded.

Eligible medical records were defined as those meeting two completeness criteria: full documentation of all variables specified in Section 2.3, and the presence of a completed and signed parental informed consent form for participation in medical research.

Vaccination status for the respective influenza season was ascertained from hospital medical records based on parental declaration documented at the time of admission. Review of all 525 eligible medical records identified no documented influenza vaccination for the respective admission season in any hospitalized child, and no parental report of influenza vaccination was recorded at admission. The clinical cohort, therefore, consisted entirely of children without documented vaccination within the available sources. It is acknowledged that this ascertainment approach, based solely on hospital medical records and parental declaration, without cross-referencing primary care records or the Romanian National Electronic Vaccination Registry (RENV), supports a finding of absence of documented vaccination rather than confirmed absence of vaccination; individual-level registry data were not accessible to hospital-based researchers under current Romanian data governance arrangements. The county-level vaccination coverage data obtained from the Galați County Public Health Authority, presented as aggregate annual counts, serve as contextual population-level epidemiological information and do not constitute individual-level validation of vaccination status, as hospitalized children may not be representative of the broader county population. This methodological constraint is acknowledged as a study limitation. The absence of any documented vaccinated child across the study period precludes within-cohort analysis of vaccine effectiveness.

**Figure 1:**
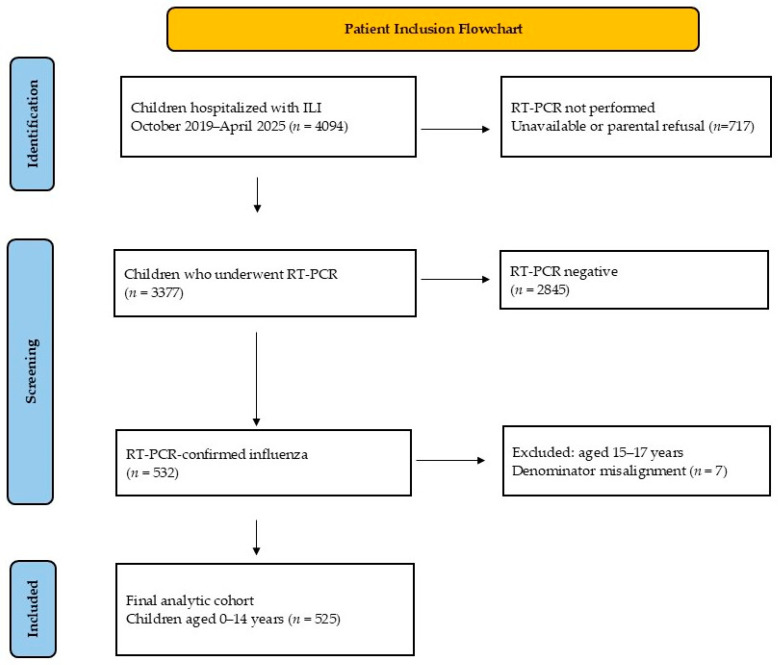
Patient inclusion flowchart. ILI = influenza-like illness; RT-PCR = reverse transcription polymerase chain reaction. Seven RT-PCR-confirmed patients aged 15–17 years were subsequently excluded from the primary analytic cohort to ensure denominator alignment with county-level vaccination coverage data reported for the 0–14 age group (final analytic cohort: *n* = 525).

Across the six study seasons, 4094 children were hospitalized with influenza-like illness; following sequential exclusions for absent RT-PCR confirmation and age restriction, the final analytic cohort comprised 525 children aged 0–14 years with RT-PCR-confirmed influenza. Full patient flow data are presented in Table 1 and Figure 1.

### 2.3. Clinical Variables

The following data were extracted from the electronic medical records: demographic characteristics (age, sex, and place of residence), admission date, presenting symptoms, laboratory-confirmed influenza type, underlying comorbidities, administered treatments, and clinical outcomes. Nutritional status was assessed using WHO growth standards, which are routinely applied in Romanian pediatric clinical practice.

Coinfections were defined and recorded separately from influenza-associated systemic manifestations. A coinfection was documented when concurrent laboratory-confirmed or clinically substantiated infection with another pathogen—including viruses (e.g., Respiratory Syncytial Virus [RSV], Severe acute respiratory syndrome coronavirus 2 [SARS-CoV-2]) or bacteria—was identified during the same admission episode. Coinfection data were reported descriptively; their association with clinical outcomes will be examined in dedicated analytical work using this cohort.

Influenza-associated systemic manifestations were categorized by organ system (respiratory, gastrointestinal [GI], neurological, hematological, renal, and cardiovascular) based on clinical diagnoses documented in the medical record. This categorization was intentionally broad, encompassing the full spectrum of organ-system involvement—from subclinical laboratory abnormalities to clinically overt disease—to provide a comprehensive epidemiological description of multisystem involvement. It did not imply that all documented findings represented severe or life-threatening disease.

Outcome variables included antiviral therapy details, requirement for supplemental oxygen or ventilatory support, admission to high-dependency monitoring, and length of hospital stay (LOS).

### 2.4. Regional Vaccination Coverage Data

Regional influenza vaccination coverage data for Galați County were obtained from the Galați County Public Health Authority (Direcția de Sănătate Publică Galați), which collects annual influenza vaccination reports from all healthcare providers in the county. Data were available for each calendar year from 2019 through 2025, disaggregated by age group (0–2 years, 3–6 years, 7–14 years, and 15 years and above), sex, urban/rural residence, and, separately, pregnant women.

Population denominators for coverage calculations were obtained from the Romanian National Institute of Statistics (Institutul Național de Statistică) TEMPO online database, which provides annual resident population estimates for Galați County disaggregated by single year of age, sex, and urban/rural residence. Coverage was calculated as the number of vaccinated individuals in each age group divided by the resident population in that age group, expressed as a percentage.

It is important to note that the vaccination data use calendar years (January–December), while the clinical hospitalization data use influenza seasons (October–April). For descriptive contextual purposes, each calendar year of vaccination data was considered most relevant to the influenza season that began in October of that year (e.g., 2022 vaccination data is presented alongside the 2022–2023 season). This alignment was an approximation and is acknowledged as a limitation.

### 2.5. High-Dependency Monitoring

At “Sf. Ioan” Clinical Emergency Pediatric Hospital in Galați, a structurally separate high-dependency unit (HDU) is not available. Children requiring close clinical monitoring—including those with respiratory distress, hemodynamic instability, altered consciousness, or a clinical trajectory warranting intensive observation—are admitted to the ICU (Intensive Care Unit), which provides a substantially higher nurse-to-patient ratio compared to general pediatric wards. This arrangement reflects local staffing and infrastructure conditions rather than a formal protocolized ICU admission threshold.

Accordingly, ICU admission in this study reflected the clinical need for close observation and enhanced nursing supervision and should not be interpreted as indicating invasive critical care interventions, such as mechanical ventilation or vasopressor support, in an exclusive manner. This practice is consistent with models reported in resource-constrained healthcare settings [24] and is acknowledged as a limitation that affects the direct comparability of ICU admission rates with data from settings with dedicated HDUs.

### 2.6. Statistical Analysis

Statistical analysis was purely descriptive, consistent with the study’s objective of characterizing the epidemiological profile of the hospitalized cohort and the regional vaccination context. No inferential analyses or statistical modeling were performed, as the study design and objectives did not require hypothesis testing or predictive modeling.

Categorical variables were summarized as absolute frequencies and proportions. Continuous variables were reported as means ± standard deviations (SD) or medians with IQR, as appropriate. All analyses were conducted using SPSS version 31.0.4.0 (IBM Corp., Armonk, NY, USA).

### 2.7. Ethics

This study was approved by the Ethics Committee of “Sf. Ioan” Clinical Emergency Pediatric Hospital in Galați (protocol code 23183/24 September 2024; renewed on 14 August 2025 with protocol code 17637). The study was conducted in accordance with the Declaration of Helsinki. Written informed consent was obtained from the parents or legal guardians of all participants.

## 3. Results

This section has two components presented in parallel: Section 3.1 shows regional influenza vaccination coverage in Galați County from public health records, while Section 3.2 and Section 3.3 describe clinical and epidemiological features of the hospitalized cohort. These components share a common catchment area and study period; their co-presentation aims to contextualize hospitalization within the regional vaccination environment, not to suggest causal links between vaccination and clinical outcomes.

### 3.1. Regional Influenza Vaccination Coverage in Galați County (2019–2025)

#### 3.1.1. Overall Coverage Trends

**Figure 2:**
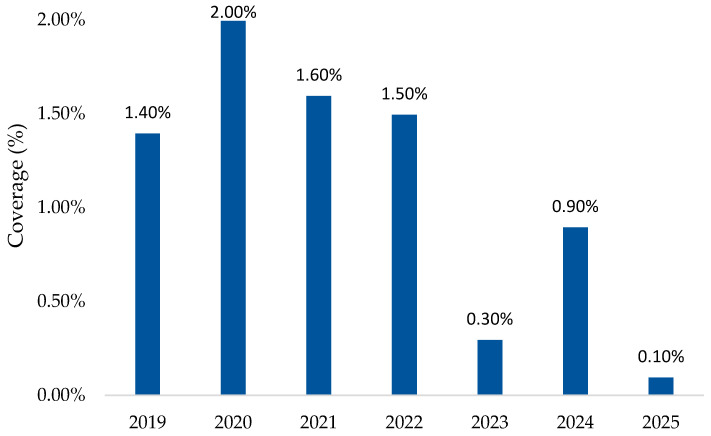
Overall influenza vaccination coverage among children aged 0–14 years, Galați County, Romania, 2019–2025.

Regional influenza vaccination coverage among children aged 0–14 years in Galați County remained consistently and critically low throughout the entire study period (Figure 2).

Total coverage across all pediatric age groups never exceeded 2.0% of the eligible resident population in any calendar year. The highest overall pediatric coverage recorded was 2.0% in 2020, corresponding to the pre-pandemic season when awareness of respiratory infections was heightened by the emerging COVID-19 pandemic. Coverage declined substantially thereafter, reaching a nadir of 0.3% in 2023—a more than sixfold decrease from the already-low 2020 peak. A partial recovery was observed in 2024 (0.9%), but coverage fell again sharply in 2025 (0.1%), the lowest value recorded across the entire study period.

#### 3.1.2. Coverage by Age Group

**Figure 3:**
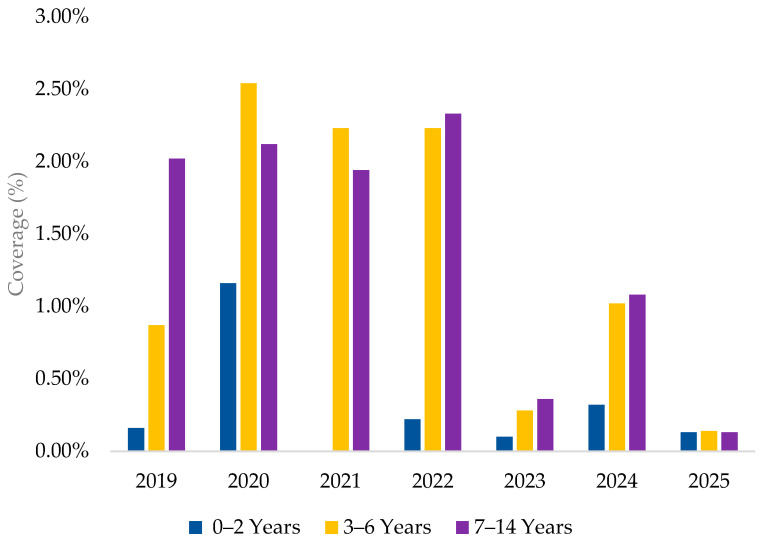
Influenza vaccination coverage by age group among children aged 0–14 years, Galați County, Romania, 2019–2025.

The youngest children—aged 0–2 years, who also constituted the largest hospitalized age group in the clinical cohort—were consistently the least vaccinated (Figure 3).

Coverage in this group peaked at 1.2% in 2020, was 0.0% in 2021, and declined to 0.1% by 2025. Coverage in the 3–6 year age group similarly peaked in 2020 (2.5%) and fell to 0.1% by 2025. The 7–14 year age group showed somewhat higher absolute numbers of vaccinated children in most years, but coverage remained very low, peaking at 2.3% in 2022 and collapsing to 0.1% in 2025.

#### 3.1.3. Urban-Rural Distribution

Vaccination was more concentrated in urban areas throughout the study period. In 2019, for example, all 27 vaccinated infants (0–2 years) were from urban areas, with none in rural areas at this age. In subsequent years, rural vaccination remained consistently lower across all age groups, with the urban–rural disparity most pronounced in the 0–2 and 3–6 year age groups.

#### 3.1.4. Pregnant Women

**Figure 4:**
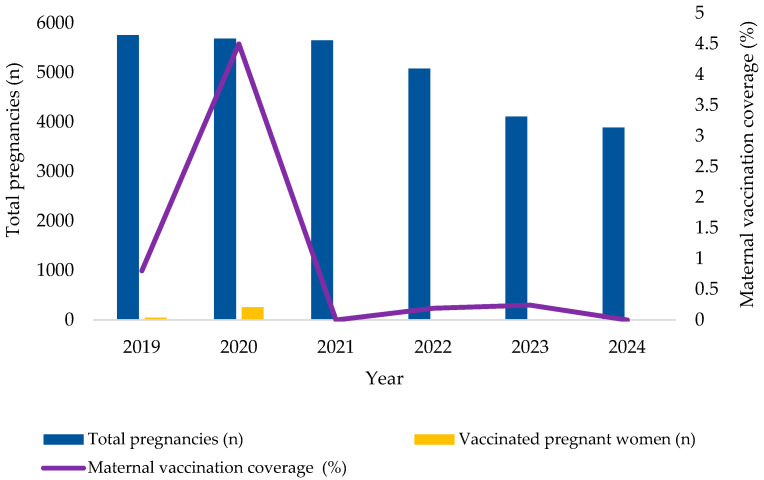
Annual number of total pregnancies, influenza-vaccinated pregnant women, and maternal influenza vaccination coverage (%) in Galați County, Romania, 2019–2024. No vaccinations were recorded in 2021 or 2024. Data for 2025 were unavailable at the time of analysis.

Vaccination of pregnant women, relevant as a strategy for indirect protection of infants too young to be immunized, was documented only sporadically across the study period. As illustrated in Figure 4, maternal influenza vaccination coverage never exceeded 4.6% of the total annual pregnancy count in any study year, with this peak recorded in 2020. In all subsequent seasons, coverage fell to below 0.5%, and no vaccinated pregnant women were recorded in 2021 or 2024.

### 3.2. Clinical Cohort—Baseline Characteristics

Baseline characteristics of the hospitalized cohort are presented in Table 2.

#### 3.2.1. Demographic Characteristics

**Figure 5:**
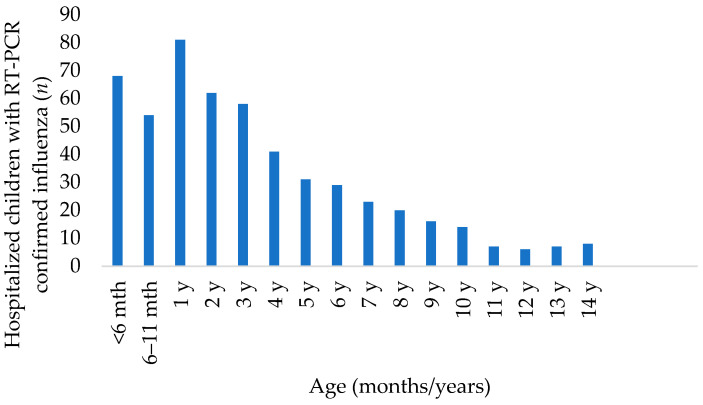
Age distribution of children hospitalized with RT-PCR-confirmed influenza, Galați County, Romania, 2019–2025 (*N* = 525) (mnth—months; y—years).

A total of 525 children aged 0–14 years hospitalized with RT-PCR-confirmed influenza between October 2019 and April 2025 were included. The median age was 2 years (IQR 1–5), with a mode of 0 years, reflecting a high proportion of infants. Children aged under 2 years constituted the largest age group (50.5%), followed by those aged 3–6 years (30.3%) and 7–14 years (19.2%). Further granular analysis of the youngest age group revealed that 68 infants (12.9%) were aged under 6 months, while 54 infants (10.3%) were aged 6–11 months, representing the youngest subgroup eligible for direct vaccination. Children under 6 months had missed maternal vaccination (Figure 5).

A slight male predominance was observed, with boys accounting for 54.1% of cases. The majority of hospitalized children (58.9%) resided in urban areas.

#### 3.2.2. Temporal Trends and Seasonality

Hospitalization data are reported by epidemiological season (October–April), while regional vaccination coverage and confirmed case notifications from the county public health authority are aggregated by calendar year; these temporal frameworks are therefore not directly equivalent and are presented in parallel for contextual purposes only.

The number of influenza-associated hospitalizations varied markedly across the study period. During the 2019–2020 season, 79 cases were recorded. The 2020–2021 season, characterized by stringent non-pharmaceutical interventions including mask mandates, school closures, and movement restrictions, yielded a single influenza hospitalization, consistent with the near-total suppression of influenza circulation documented nationally during this period. The divergence between zero county-level sentinel surveillance notifications and the RT-PCR-confirmed hospitalizations recorded at the study institution during the same period is addressed in Section 4. Hospitalization activity remained historically low in 2021–2022 (21 cases), reflecting the continued attenuation of influenza transmission amid residual pandemic-era behavioral changes. Following the progressive relaxation of public health restrictions, hospitalizations increased substantially: the 2022–2023 season recorded 158 cases, exceeding pre-pandemic levels. Elevated activity persisted in 2023–2024 (128 cases) and 2024–2025 (138 cases).

**Figure 6:**
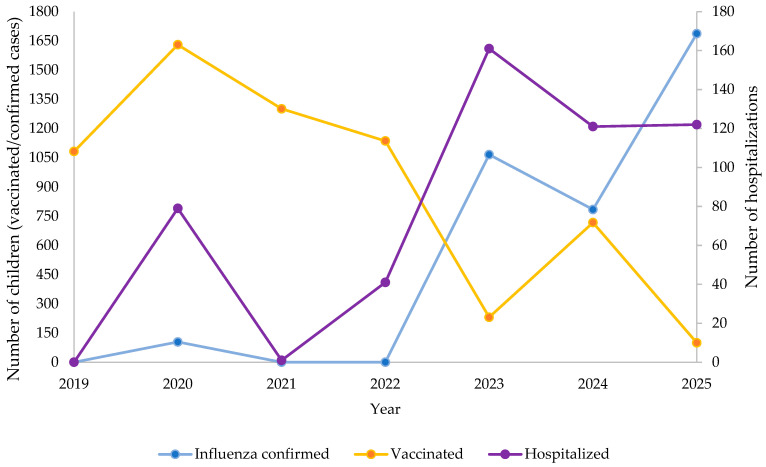
Temporal trends in influenza vaccination coverage, laboratory-confirmed influenza notifications, and pediatric influenza-associated hospitalizations in Galați County, Romania, across the study period (2019–2025). Hospitalization counts are displayed on the secondary axis (right).

The temporal relationship between regional vaccination numbers and hospitalization counts across the study period is illustrated in Figure 6. Vaccination numbers among children aged 0–14 years declined progressively from 2022 onward, reaching the lowest recorded value in 2025 (*n* = 100 county-wide), while hospitalization activity remained persistently elevated across the three post-pandemic seasons, peaking in 2022–2023 (158 cases). This temporal co-occurrence is presented as contextual epidemiological framing; the descriptive design of this study precludes causal inference. These temporal patterns mirror those reported at the national level. National surveillance data indicate that children consistently represented one of the most heavily affected demographic groups during seasonal influenza in Romania, accounting for approximately 30–40% of all influenza-like illness (ILI) cases in pre- and post-pandemic seasons [12,13,14,15,16]. At the regional level, Galați County recorded the highest number of cases in 2025 (1687) followed by 2023 (1066) and 2024 (784). This provides important context for the hospitalized cohort described in this study.

**Figure 7:**
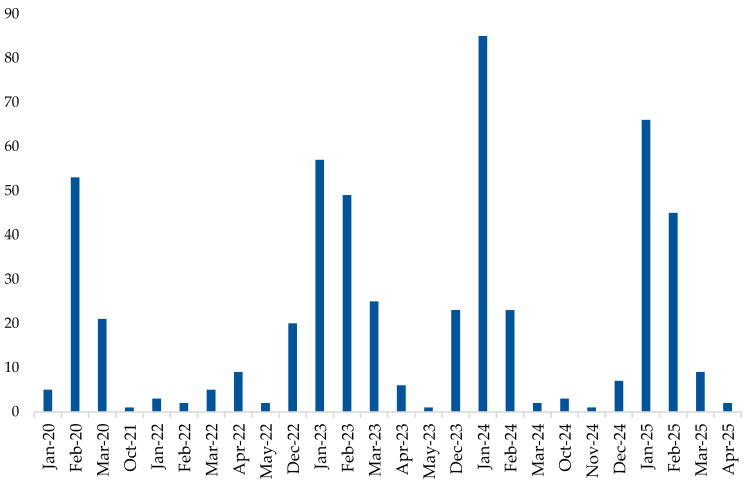
Monthly frequency distribution of children hospitalized with RT-PCR-confirmed influenza, Galați County, Romania, 2019–2025. Missing months across the seasons had no admissions.

Clear seasonal patterns re-emerged from 2022 onward, with peaks consistently occurring during January–March of each season (Figure 7). January 2024 recorded the highest monthly case count, with more than 80 hospitalizations. Winter-season hospitalization numbers in the post-pandemic period consistently exceeded those observed before the COVID-19 pandemic.

#### 3.2.3. Influenza Virus Types

**Figure 8:**
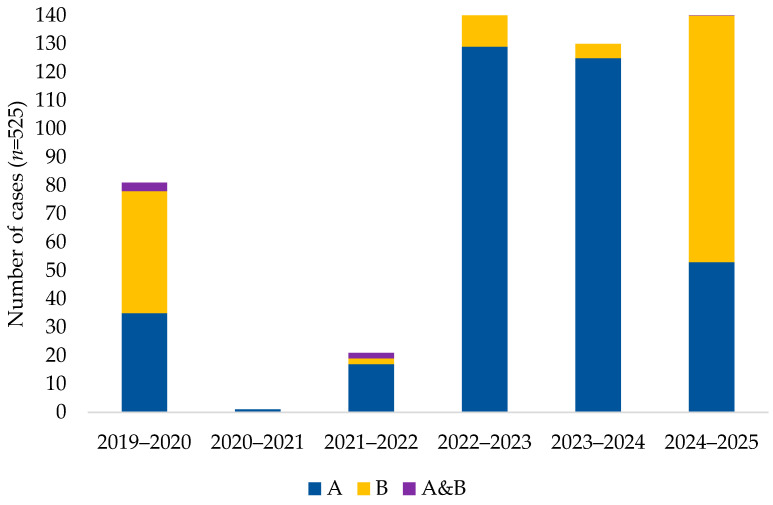
Distribution of influenza virus types (A, B, and A&B co-detection) by season among children hospitalized with RT-PCR-confirmed influenza, Galați County, Romania, 2019–2025.

RT-PCR testing confirmed influenza infection in all cases; however, consistent subtype or lineage determination was not performed at the study institution. Influenza A was identified in 67.8% of cases, influenza B in 31%, and co-detection of both types in 1.2%. The seasonal distribution of influenza virus types is illustrated in Figure 8.

#### 3.2.4. Clinical Presentation

Children hospitalized with influenza presented with a broad spectrum of symptoms, stratified by age group in Table 3.

The most frequent presenting symptom was fever (91.2%), which was commonly the primary reason for hospital presentation. Cough (76.8%) and nasal congestion or rhinorrhea (70.5%) were the next most common features. Fatigue or malaise was reported in 69.1% of patients. Pharyngeal inflammation was present in nearly half of the cohort (48.2%), and signs of respiratory distress—including tachypnea, retractions, and increased work of breathing—were documented in 44.2%.

Gastrointestinal symptoms were frequent: abdominal pain in 38.7%, vomiting in 29.9%, and diarrhea in 18.7%. Neurological manifestations were less common but clinically notable; headache was reported in 5.3% of cases and seizures in 5.1%. Myalgia (5.1%), rash (2.9%), and confusion or altered mental status (1.1%) were infrequently documented.

### 3.3. Hospitalization Data

#### 3.3.1. Hospital Course and Treatments

The majority of patients (67.24%) had no documented underlying medical condition. Underlying comorbidities were present in 32.76% of patients (Table A1, Appendix A). Nutritional disorders were most prevalent: failure to thrive or undernutrition (13.14%), underweight (5.33%), and obesity (3.81%). Classic high-risk conditions—asthma and epilepsy (each 0.76%) and malignancy or immunodeficiency (each < 1%)—were uncommon.

All patients received supportive care. Oseltamivir was administered to the vast majority; the median time from admission to initiation was 1 day (mean 2.3 ± 2.68 days). Antibiotics were prescribed in 62.7% of cases. Confirmed bacterial coinfections were most commonly attributable to Streptococcus pneumoniae or Staphylococcus aureus. Viral coinfections were less frequent, with SARS-CoV-2 and RSV as the most commonly co-detected pathogens. Coinfection data are reported here for descriptive completeness; their relationship with clinical outcomes will be examined in dedicated analytical publications.

#### 3.3.2. Influenza-Associated Systemic Manifestations

Multisystem clinical involvement was documented in 97.52% of patients (Table A2, Appendix A). As described in Section 2.3, this figure reflects broad ascertainment across the full severity spectrum and should not be interpreted as indicating that the majority of patients experienced severe or life-threatening events.

Respiratory involvement was documented in 455 patients (86.66%): 232 (44.19%) had lower respiratory tract involvement, 115 (21.9%) had upper respiratory tract involvement, and 108 (20.57%) had concurrent lower and upper tract disease. Pneumonia was the most frequent lower respiratory tract finding (38.47%); pharyngotonsillitis was the most common upper tract finding (17.52%).

Hematological findings—including anemia, cytopenias, and coagulopathy across varying degrees of severity—were documented in 80.19% of admissions. These findings almost invariably co-occurred with respiratory involvement; isolated hematological findings in the absence of other organ-system involvement were rare, accounting for 0.76% of cases.

Gastrointestinal findings (severe dehydration, electrolyte imbalance, or elevated hepatic enzymes) were documented in 45.52% of patients in combination with other system involvement, and rarely as isolated findings (4.76%). Neurological manifestations were clinically significant: seizures occurred in 5.14% and encephalopathy or confusion in 1.14% of patients. Renal and cardiac involvement were rare findings.

#### 3.3.3. Outcomes

Key hospitalization outcomes are summarized in Table 4.

Despite widespread multisystem involvement, only 91 patients (17.3%) required high-dependency monitoring, and stays were generally brief (approximately 15% of all patients spent 1–3 days in the high-dependency unit). As described in Section 2.5, this admission reflects clinical need for close monitoring in the absence of a separate HDU and is not equivalent to invasive critical care. Supplemental oxygen was required in 35.4% of patients, with a mean duration of 0.93 ± 1.61 days across the cohort; 75.26% of those requiring oxygen needed it for 1–3 days only. Mechanical ventilation was infrequently required; no patient required extracorporeal membrane oxygenation (ECMO).

**Figure 9:**
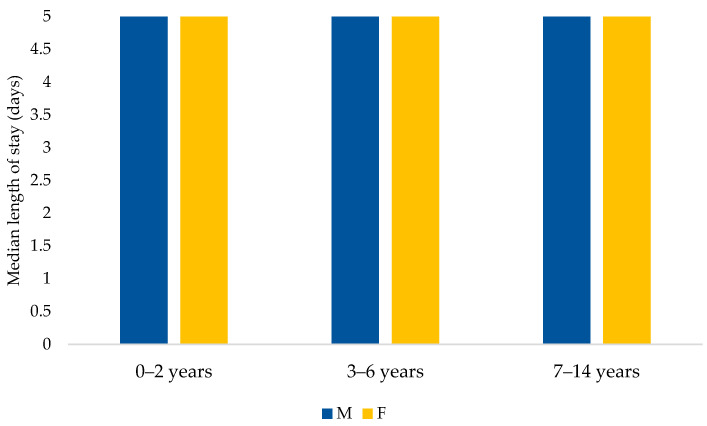
Median length of hospital stay (days) by age group and sex in children hospitalized with RT-PCR-confirmed influenza, Galați County, Romania, 2019–2025.

The mean hospital stay was 5.93 ± 3.14 days (median 5; IQR 4–7) (Figure 9). Fewer than 10% required stays exceeded 10 days.

All patients recovered with supportive management. No in-hospital deaths were recorded across the entire six-season study period in the 0–14 years age group.

## 4. Discussion

This retrospective descriptive study presents, to the best of the authors’ knowledge, the first published pairing of a multi-season clinical cohort of hospitalized pediatric influenza patients with granular regional vaccination coverage data from the same catchment area in Romania. Both components yielded a consistent picture: influenza vaccination coverage among children in Galați County was critically and persistently low across all six study seasons, and the entire hospitalized cohort in the parallel clinical series was unvaccinated. Notably, this finding emerged from retrospective records review rather than pre-specified inclusion criteria, and is consistent with the critically low population-level coverage documented in the regional surveillance data.

The regional vaccination coverage data are striking in their uniformity of inadequacy. Total pediatric coverage (ages 0–14) never exceeded 2.0% in any year, and the most recent seasons of the study recorded some of the lowest values—0.3% in 2023 and 0.1% in 2025. This places Galați County at the very low end of the already low European pediatric vaccination coverage spectrum [14]. The collapse in 2023 is particularly notable: across all age groups, absolute vaccination numbers fell to a small fraction of prior seasons, coinciding temporally with sustained high influenza hospitalization activity documented in the clinical cohort from the 2022–2023 epidemiological season onward. While this temporal parallel cannot establish causation in a descriptive study—and noting that the absence of a vaccinated comparator group and the lack of population-level incidence rates preclude any direct inference between vaccination coverage levels and hospitalization burden—it is presented as a contextual epidemiological observation consistent with the broader literature on vaccination coverage and influenza burden [25,26,27].

The youngest children—aged 0–2 years—were the least vaccinated group in every study season, with coverage in this group never exceeding 1.2% and falling to effectively zero by 2025. This is particularly concerning because this age group simultaneously represented the largest hospitalized subgroup (50.5% of the clinical cohort). The convergence of maximal clinical vulnerability—attributable to immunological immaturity and limited prior antigen exposure [4,28,29,30]—with minimal vaccine protection represents a critical gap in preventive care for this population.

The near-complete absence of maternal influenza vaccination is an equally important finding. Vaccinated pregnant women numbered fewer than 50 county-wide in all seasons except 2020, and no vaccinated pregnant women were recorded at all in 2021, 2024, or 2025. Maternal influenza vaccination coverage peaked at 4.6% in 2020, the sole season in which uptake across all target groups was transiently elevated, before declining to below 0.5% in all subsequent seasons. The regional pregnancy count declined from approximately 5700 in 2019 to approximately 3850 in 2024, consistent with documented demographic trends in the county form the Romanian National Institute of Statistics TEMPO online database; maternal vaccination coverage remained below 0.5% throughout this period regardless of the declining denominator. Given that infants aged 0–5 months are ineligible for direct influenza vaccination and are dependent on transplacentally transferred maternal antibodies for vaccine-derived protection [9,31], the virtual absence of maternal immunisation across most study seasons represents a critical and unaddressed gap in indirect infant protection in this setting.

The urban–rural disparity in vaccination uptake adds a further dimension of inequality. Rural children and pregnant women were consistently less vaccinated than their urban counterparts across all age groups and all years. This pattern likely reflects a combination of reduced healthcare access, fewer primary care touchpoints for vaccine delivery, lower health literacy, and reduced community awareness in rural settings [32]—barriers that targeted outreach programs could address.

These findings are consistent with data from comparable low-vaccination settings in Eastern and Southern Europe. Studies from Poland, Italy, and other European countries have similarly documented that low pediatric influenza vaccination coverage is associated with disproportionate hospitalization burden in young, otherwise healthy children [28,33]. A Romanian study of hospitalized influenza patients during the 2019–2020 season confirmed H1N1 predominance and highlighted the vulnerability of unvaccinated pediatric populations in the region [34]. More broadly, European surveillance data consistently show that countries with the lowest vaccination coverage rates experience the highest rates of influenza-associated hospitalization in children, reinforcing the public health case for strengthened pediatric vaccination programs [13,25]. The regional data presented in this study—derived from the sole pediatric emergency hospital serving Galați County, to which all pediatric influenza cases requiring inpatient management are referred—demonstrate vaccination coverage below 2% throughout the study period in a county of 496,892 residents, representing among the lowest documented pediatric influenza vaccination rates in the published European literature. It should be noted, however, that the cohort captures only hospitalised cases and therefore represents a selected subset of the total pediatric influenza burden in the region; mild and moderate cases managed in primary or community care settings are not represented.

The clinical cohort characteristics are described in the context of this low-vaccination environment. The median age of 2 years and the concentration of cases in children under 5 are consistent with European data [28,33]. A slight male predominance (54.1%) was observed, consistent with reported sex-based differences in susceptibility to acute respiratory tract infections in childhood [35]. Notably, two-thirds of patients were previously healthy—a pattern diverging from U.S. surveillance data where 70–80% of hospitalized pediatric influenza patients have underlying comorbidities [4,36], and consistent with findings from other low-vaccination settings where healthy unvaccinated children constitute the majority of severe cases [9,25,26,31]. This descriptive observation did not establish causal attribution; however, it was consistent with the established evidence that influenza vaccination effectively reduces hospitalization risk in both healthy and high-risk children [26,27].

The temporal distribution of hospitalizations mirrors regional vaccination trends. The 2020–2021 season—the only year with notably higher vaccination numbers in the data (driven largely by pandemic-related awareness)—also coincided with near-complete suppression of influenza circulation due to non-pharmaceutical interventions [37,38]. From 2022 onward, as vaccination coverage fell sharply, hospitalization numbers exceeded pre-pandemic levels and remained elevated through 2024–2025. The mechanisms underlying post-pandemic influenza rebounds in young children have been discussed extensively in the literature [38,39,40,41]; however, this study lacks the serological or individual-level exposure data required to evaluate these mechanisms, and the temporal trends are presented solely as descriptive epidemiological context.

A notable discrepancy exists between the county-level laboratory-confirmed influenza case notifications obtained from the Galați County Public Health Authority and the RT-PCR-confirmed hospitalization data presented in this study. During the 2019 (corresponding to the study’s initial partial season), 2021, and 2022 calendar years, the county sentinel surveillance system recorded zero confirmed influenza cases, whereas the clinical cohort contributed 81, 1, and 21 hospitalizations during the corresponding epidemiological seasons. This discrepancy reflects a well-documented structural limitation of passive sentinel surveillance systems, which capture only a subset of cases identified through designated reporting networks and are inherently subject to underascertainment—particularly under conditions of reduced healthcare-seeking behavior, altered testing practices, and disrupted reporting pathways [15,16,17,18,19]. During the 2020–2022 period, pandemic-era non-pharmaceutical interventions substantially suppressed both influenza transmission and surveillance sensitivity nationally, as evidenced by near-zero confirmed case counts reported across Romania during the same interval [15,16]. Hospital-based RT-PCR confirmation, by contrast, operates through a distinct and independent ascertainment pathway, capturing cases that present with sufficient clinical severity to warrant hospitalization regardless of whether they are subsequently notified through the sentinel system. The divergence between these two data streams underscores the limitations of county-level surveillance data as a comprehensive measure of true influenza burden and reinforces the value of hospital-based cohort data in characterizing clinical and epidemiological patterns in settings where population-level surveillance remains incomplete.

The virological profile—two-thirds influenza A and one-third influenza B—is typical for severe pediatric influenza cohorts [42]. Both types caused a significant hospitalization burden, consistent with comparative studies demonstrating clinically meaningful disease attributable to both influenza A and B in pediatric populations [43,44], and supporting the continued use of quadrivalent vaccines covering both A subtypes and B lineages, as well as routine testing for both types in hospitalized children.

Oseltamivir was administered to the vast majority of patients, consistent with current guidelines recommending early antiviral therapy for hospitalized children with confirmed influenza [45,46,47]. Antibiotic use was high (62.7%), consistent with the clinical difficulty of distinguishing influenza-associated lower respiratory tract involvement from bacterial superinfection [48].

The descriptive characterization of coinfections in this cohort provides a foundation for future analytical work; however, no inferential conclusions regarding their association with disease severity or clinical outcomes are drawn in the present study, consistent with the purely descriptive design. The prevalence and clinical impact of bacterial and viral coinfections in pediatric influenza have been documented across multiple settings [49,50,51,52,53,54].

Multisystem clinical involvement was documented in 97.52% of patients. As detailed in the Methods, this reflected broad ascertainment of organ-system findings across the full severity spectrum—from subclinical laboratory abnormalities to clinically overt disease—and should not be read as indicating that nearly all patients experienced severe illness. Respiratory involvement was the predominant finding (86.66%), with pneumonia the most frequent. Severe respiratory compromise, including acute respiratory distress syndrome, has been documented as an uncommon but clinically significant complication of pediatric influenza [55]. Hematological findings—including anemia, cytopenias, and coagulopathy across varying degrees of severity—were documented in 80.19% of admissions, consistent with reports of hematological involvement in pediatric influenza across diverse settings [56,57]. Hepatic enzyme elevation, a recognized albeit less frequently reported manifestation, has similarly been documented in pediatric influenza cohorts [58]. The predominance of respiratory findings and pneumonia as the leading complication is consistent with the established severity profile of hospitalized pediatric influenza [59,60,61]. Neurological events—including seizures and encephalopathy—were documented in a clinically important subset, consistent with global reports of influenza-associated neurological events [62,63]. Cardiac and renal findings, though rare, were noted and merit clinical vigilance in similar settings [64].

Despite widespread multisystem involvement, high-dependency monitoring was required in only 17.3% of patients and stays were brief—reflecting both the secondary-level nature of the institution and the absence of a separate HDU, as described in the Methods. All children included in this cohort were safely discharged, indicating that with timely clinical recognition and appropriate supportive management, outcomes can be favorable [37,65,66,67,68].

## 5. Limitations

This study has several limitations. As a retrospective, descriptive, single-center study at a secondary pediatric hospital, the clinical cohort likely represents a more severely ill subset of the pediatric influenza population; findings are not generalizable to the broader community or primary care settings. Population-based incidence rates could not be calculated as age-stratified denominator data for the hospital’s catchment area were unavailable for the full study period.

Vaccination status was ascertained solely from parental declaration documented in hospital medical records, without cross-referencing primary care records or the Romanian National Electronic Vaccination Registry (RENV). Individual-level registry access was not available under the current data governance arrangements. This ascertainment approach supports a finding of absence of documented vaccination within available sources rather than confirmed absence of vaccination, and constitutes a methodological limitation of the study.

The vaccination coverage data represent county-level aggregate totals used exclusively as contextual epidemiological framing and were not intended for individual-level linkage with the clinical cohort. The two components of this study, the clinical cohort and the regional coverage data, are presented in parallel as descriptive data streams consistent with the study’s design. The temporal alignment of calendar-year vaccination data with influenza-season hospitalization data is an approximation, as described in Section 2.4. Coverage estimates for the 0–2 years age group may be influenced by changes in birth cohort size across the study period, as reflected in the declining resident population in this group.

The regional vaccination coverage data were available for children aged 0–14 years, while the clinical cohort included patients up to 17 years of age. The age restriction to 0–14 years was applied to ensure denominator alignment with county-level vaccination coverage statistics reported by the Galați County Public Health Authority for the 0–14 age group. Patients aged 15–17 years accounted for 1.3% of the hospitalized population (7 of 532 patients) and were accordingly excluded from the primary analytic cohort. One in-hospital death was recorded among the excluded 15–17 age subgroup; this patient is not represented in the outcome data reported herein. This exclusion is acknowledged as a limitation, particularly regarding the completeness of the mortality data.

The broad ascertainment of organ-system involvement limits direct comparison with studies that apply more restrictive definitions. ICU admission criteria were not formally protocolized and reflected local staffing constraints, as described in Section 2.5. Vaccine status in the clinical cohort was determined from medical documentation and parental reporting, which may be subject to minor recall bias. Influenza subtype and lineage data were not available. The descriptive design of this study precludes analytical assessment of predictors of disease severity or the clinical impact of coinfections; these questions remain open for future investigation.

## 6. Conclusions

Influenza vaccination coverage among children aged 0–14 years in Galați County was persistently negligible throughout the study period, never exceeding 2.0% in any year and falling to 0.1% by 2025. Infants and toddlers—the most clinically vulnerable group and the largest hospitalized subgroup—were consistently the least vaccinated. Maternal influenza vaccination, a key strategy for indirect infant protection, was effectively absent in most seasons.

In the parallel clinical cohort, hospitalizations were dominated by previously healthy infants and preschool-aged children; multisystem clinical involvement was widespread. All patients in the 0–14 years analytic cohort were successfully discharged following supportive management.

These regional descriptive data document a critical and persistent gap in pediatric and maternal influenza vaccination in Galați County, contextualized against a hospitalization burden concentrated in the youngest and most immunologically vulnerable age groups. As two independently described data streams presented within the same geographic and temporal context, these findings provide regionally grounded descriptive evidence to inform targeted vaccination program development—through enhanced primary care engagement, rural outreach, and sustained public awareness—in this and similar low-coverage Eastern European settings.

## Figures and Tables

**Table 1:** Seasonal distribution of influenza-like illness hospitalizations, RT-PCR testing outcomes, and laboratory-confirmed influenza cases across six consecutive epidemiological seasons, “Sf. Ioan” Clinical Emergency Pediatric Hospital, Galați County, Romania, 2019–2025.

Season	ILI (*n*)	No RT-PCR (*n*)	PCR Negative (*n*)	Confirmed (*n*)
2019–2020	626	307	240	79
2020–2021	115	0	114	1
2021–2022	583	0	562	21
2022–2023	778	0	620	158
2023–2024	893	136	629	128
Total	4094	717	2845	532

**Table 2:** Baseline characteristics of patients hospitalized with RT-PCR-confirmed influenza (*N* = 525).

	*N* (%)
Sex	
Male	284 (54.1%)
Female	241 (45.9%)
Age groups	
0–2 years	265 (50.5%)
3–6 years	159 (30.3%)
7–14 years	101 (19.2%)
Residence	
Urban	309 (58.9%)
Rural	216 (41.1%)
Influenza virus strain	
A	356 (67.8%)
B	163 (31.1%)
A&B	6 (1.1%)
Underlying Comorbidities	
None	353 (67.2%)
Present	172 (32.8%)
Influenza vaccination	
No	525 (100%)
Yes	0 (0%)

**Table 3:** Presenting symptoms in children hospitalized with RT-PCR-confirmed influenza (*N* = 525).

Symptom	*N* (Estimate)
Fever	479 (91.2%)
Cough	403 (76.8%)
Nasal congestion/rhinorrhea	370 (70.5%)
Fatigue/malaise	363 (69.1%)
Pharyngeal inflammation	253 (48.2%)
Respiratory distress	232 (44.2%)
Abdominal pain	203 (38.7%)
Vomiting	157 (29.9%)
Diarrhea	98 (18.7%)
Headache	28 (5.3%)
Myalgia	27 (5.1%)
Seizures	27 (5.1%)
Rash	15 (2.9%)
Confusion/altered mental status	6 (1.1%)

**Table 4:** Hospitalization outcomes and resource utilization.

Parameter	Mean ± SD or *n* (%)	Median [IQR]
High-dependency monitoring admission	91 (17.3%)	—
High-dependency monitoring duration (days)	0.46 ± 1.24	0 [0–0]
Supplemental oxygen required	186 (35.4%)	—
Oxygen therapy duration (days)	0.93 ± 1.61	0 [0–1]
Hospital length of stay (days)	5.93 ± 3.14	5 [4–7]

Continuous variables are expressed as mean ± standard deviation or median (interquartile range) as appropriate to data distribution; categorical variables are expressed as absolute frequency and proportion (%).

## Data Availability

The data supporting the reported results in this study originate from the internal electronic database of the “Sf. Ioan” Clinical Emergency Pediatric Hospital in Galați, and are not publicly available due to ethical and privacy restrictions. Researchers interested in accessing anonymized data may submit a request to the corresponding author, in accordance with Ethics Committee approval.

## Abbreviations

The following abbreviations are used in this manuscript:

RT-PCR	Reverse Transcriptase Polymerase Chain Reaction
IQR	Interquartile Range
WHO	World Health Organization
COVID-19	Coronavirus disease 2019
SARS-CoV-2	Severe acute respiratory syndrome coronavirus 2
ECDC	European Center for Disease Prevention and Control
RSV	Respiratory Syncytial Virus
GI	Gastrointestinal
LOS	Length of hospital stay
HDU	High-dependency unit
ICU	Intensive Care Unit
SD	Standard deviation
ECMO	Extracorporeal Membrane Oxygenation
SARS-CoV-2	Severe Acute Respiratory Syndrome Coronavirus
RNA	Ribonucleic acid

## Appendix A

**Table A1:** Patients comorbidities.

Comorbidities	Frequency	Percent
No comorbidities	353	67.24
Failure to thrive	69	13.14
Underweight	28	5.33
Obesity	20	3.81
Cardiac malformation, Failure to thrive	5	0.95
Protein-energy malnutrition	5	0.95
Asthma	4	0.76
Cardiac malformation, Protein-Energy Malnutrition	4	0.76
Diabetes	4	0.76
Epilepsy	4	0.76
Spastic tetraparesis, Protein-Energy Malnutrition	2	0.38
Acute lymphoblastic leukemia	1	0.19
Asthma, Failure to thrive	1	0.19
Immune Thrombocytopenia	1	0.19
Autoimmune thyroid disease	1	0.19
Cardiac malformation, Cardiac failure, Protein-Energy Malnutrition	1	0.19
Cerebral and renal tumor, Chemotherapy-induced immunosuppression, Protein-Energy Malnutrition	1	0.19
Cerebral tumor, Chemotherapy-induced immunosuppression, Protein-Energy Malnutrition	1	0.19
Chronic Kidney Disease, Protein-Energy Malnutrition	1	0.19
Congenital hydrocephalus, Obesity	1	0.19
Congenital hydrocephalus, Failure to Thrive	1	0.19
Crohn’s Disease	1	0.19
Di George Syndrome	1	0.19
Dravet syndrome, Protein-Energy Malnutrition	1	0.19
Duchenne Muscular Dystrophy, Protein-Energy Malnutrition	1	0.19
Encephalopathy, Epilepsy, Protein-Energy Malnutrition	1	0.19
Epilepsy, Spastic Tetraparesis, Protein-Energy Malnutrition	1	0.19
Gastric Ulcer	1	0.19
Glanzmann Thrombasthenia	1	0.19
Hepatitis B	1	0.19
Hurler syndrome, Protein-Energy Malnutrition	1	0.19
Hypertension, Obesity	1	0.19
Juvenile Idiopathic Arthritis	1	0.19
Marshall Syndrome	1	0.19
Failure to Thrive, Spinal Muscular Atrophy Type 1	1	0.19
Portal thrombosis, Underweight	1	0.19
Pulmonary malformation, Cerebral atrophy, Spastic Tetraparesis, Protein-Energy Malnutrition	1	0.19
Recurrent Wheezing Syndrome	1	0.19

**Table A2:** Multisystemic manifestations.

Systemic Manifestations	Frequency	Percent
Respiratory + Hematological	174	33.14
Respiratory + Hematological + Gastrointestinal	137	26.1
Respiratory	29	5.52
Gastrointestinal	25	4.76
Respiratory + Gastrointestinal	24	4.57
Respiratory + Cardiovascular & Neuromuscular + Hematological	23	4.38
Respiratory + Hematological + Neurological	18	3.43
No complications	13	2.48
Respiratory + Cardiovascular & Neuromuscular + Hematological + Gastrointestinal	11	2.10
Respiratory + Hematological + Neurological + Gastrointestinal	10	1.90
Cardiovascular & Neuromuscular + Hematological + Gastrointestinal	8	1.52
Hematological + Gastrointestinal	8	1.52
Respiratory + Hematological + Renal + Gastrointestinal	7	1.33
Respiratory + Hematological + Renal	6	1.14
Cardiovascular & Neuromuscular + Hematological	4	0.76
Hematological	4	0.76
Respiratory + Cardiovascular & Neuromuscular	4	0.76
Hematological + Neurological	3	0.57
Hematological + Neurological + Gastrointestinal	3	0.57
Respiratory + Cardiovascular & Neuromuscular + Gastrointestinal	3	0.57
Respiratory + Cardiovascular & Neuromuscular + Hematological + Renal	3	0.57
Respiratory + Cardiovascular & Neuromuscular + Hematological + Renal + Gastrointestinal	1	0.19
Cardiovascular & Neuromuscular	1	0.19
Cardiovascular & Neuromuscular + Renal	1	0.19
Cardiovascular & Neuromuscular + Neurological	1	0.19
Renal	1	0.19
Respiratory + Renal + Gastrointestinal	1	0.19
Respiratory + Neurological + Gastrointestinal	1	0.19
Respiratory + Neurological + Hematological + Renal	1	0.19
